# Macro- and metabolome-based characterization between gut microbiota and metabolites in patients with colorectal adenomas

**DOI:** 10.3389/fmicb.2025.1628315

**Published:** 2025-10-21

**Authors:** Guo Zhili, Liu Jie, Yin Peihao

**Affiliations:** ^1^Jiaxing Hospital of Traditional Chinese Medicine, Jiaxing University, Jiaxing, China; ^2^Putuo Hospital Affiliated to Shanghai University of Traditional Chinese Medicine, Shanghai, China

**Keywords:** colorectal adenoma, microbiota, metagenome, metabolomics, biomarker

## Abstract

**Objective:**

The gut microbiota has been recognized as a significant regulator in the development and progression of colorectal adenoma (CRA). However, few studies have investigated the presence and association of resident microbial species and metabolites in patients with CRA. Our aim was to analyze differences in gut microbiome composition and metabolites, as well as to evaluate their diagnostic potential for CRA.

**Methods:**

We conducted metagenomic and metabolomic analyses on fecal samples from 90 subjects, including 60 patients with CRA (CRA group) and 30 healthy subjects who served as normal controls (NC group). By integrating fecal metagenomic and metabolomic data, we identified gut microbiota-associated metabolites that showed significant abundance changes in CRA patients. Furthermore, we explored whether these metabolites and microbial species could distinguish CRA patients from healthy individuals.

**Results:**

16S rRNA gene sequencing and untargeted metabolomics analysis revealed microbial changes that distinguished CRA patients from controls. Microbial population analysis showed that the CRA group formed distinct clusters from the controls, with significant β-diversity (PCA and PCoA analyses, *p* < 0.05). At the phylum level, the dominant taxa in terms of relative abundance included Firmicutes, Ascomycota, Mycobacteria, Actinobacteria, and Clostridia. Differential analysis of the gut flora based on species abundance revealed significant differences in taxonomic composition between healthy individuals and CRA patients. KEGG functional enrichment analysis indicated that the differential flora were primarily involved in metabolic pathways, including metabolic pathways, biosynthesis of secondary metabolites, microbial metabolism in diverse environments, amino acid biosynthesis, and cofactor biosynthesis. In this study, three microbial species—*Fusobacterium mortiferum, Alistipes*, and *Bacteroides fragilis*—were validated as discriminators between healthy individuals and CRA patients, with *Alistipes* showing higher classification efficacy. Metabolomic analysis revealed differences in tryptophan metabolism, protein degradation products, amides, and phenolic acid metabolites. KEGG enrichment results indicated that metabolic pathways were the most significantly enriched. Differential metabolites were mainly associated with the biosynthesis of plant secondary metabolites. Procrustes and Venn analyses were performed on functional entries of the two omics datasets, highlighting enriched pathways including Metabolic pathways, Glycerophospholipid metabolism, Sphingolipid metabolism, and Alpha-linolenic acid metabolism. A review of the literature confirmed that the differential flora and metabolites are associated with adenoma growth.

**Conclusion:**

In this study, metagenomic and metabolomic analyses were conducted in subjects with CRA. The findings based on fecal metagenomic and metabolomic assays suggest that intestinal microecology is altered in CRA patients, leading to changes in gut cellular structure.

## 1 Introduction

CRA is a significant precancerous lesion for colorectal cancer (CRC) and its development is a multifactorial, multistep, and complex process (Sninsky et al., 2022). In recent years, advances in high-throughput sequencing and metabolomic technologies have made the role of the gut microbiota in colorectal adenomas a research focus (Ni et al., 2024). Numerous studies have shown that the gut microbiota contributes to the development and progression of CRA through various mechanisms, including metabolite production, immune regulation, and modulation of barrier function. Alterations in its composition and function are closely linked to host health (Song et al., 2024). Both domestic and international studies have consistently demonstrated significant differences in the gut microbial composition between CRA patients and healthy individuals. However, the mechanisms underlying microbiota–host interactions remain incompletely understood, particularly how microbial metabolites influence adenoma formation through host signaling pathways (Jodal et al., 2022).

Although several multi-omics studies on CRC have been conducted, they have not fully elucidated the potential involvement of microbes during the adenoma stage or the causal relationships between the microbiome/metabolome and tumorigenesis. Research on CRA and CRC has advanced considerably through the application of multi-omics approaches, particularly metagenomic and metabolomic analyses. Therefore, we prospectively collected fecal samples from individuals undergoing colonoscopy screening who were diagnosed with CRA and conducted microbiome and metabolome profiling. This study aims to clarify the role of the gut microbiome in CRA patients during CRC carcinogenesis.

The gut microbiota in CRC patients plays a crucial role in cancer initiation and progression through multiple mechanisms. Microbes contribute to both pre- and post-tumor stages by inducing genetic mutations and modulating metabolic processes. Bacterial genotoxins and metabolites can cause DNA damage in host cells, leading to genetic mutations. For example, pathogenic Escherichia coli strains carrying the polyketide synthase (pks) island induce DNA double-strand breaks and disrupt the cell cycle. Additionally, toxins produced by Pseudomonas aeruginosa elevate reactive oxygen species (ROS), resulting in DNA damage.

Studies have shown substantial alterations in the gut microbiome of CRC patients, with certain microbial species being implicated in disease progression. For instance, Fusobacterium nucleatum has been consistently associated with CRC and is considered a potential biomarker for early detection (Clos-Garcia et al., 2020; Yachida et al., 2019). Metabolomic analyses have also identified significantly elevated levels of metabolites such as branched-chain amino acids and bile acids in CRC patients (Yachida et al., 2019). One study identified a panel of gut microbiome-associated serum metabolites that accurately distinguish CRA and CRC from healthy controls, highlighting their potential as non-invasive diagnostic tools (Chen et al., 2022). Another study emphasized the role of gut microbiota and metabolites in the early stages of CRC pathogenesis, underscoring the importance of these interactions in disease development (Kim et al., 2020). Moreover, multi-omics approaches have been employed to investigate the progression from CRA to CRC. Integrated analyses of fecal metagenomics and metabolomics have revealed specific microbial and metabolic changes during this transition, providing insights into the molecular mechanisms of tumorigenesis (Clos-Garcia et al., 2020). Furthermore, the integration of metagenomics and metabolomics technologies offers new potential targets for early diagnosis and intervention (Takeuchi et al., 2024). Research suggests that modifying the gut microbiota composition or supplementing with specific metabolites, such as butyrate, may represent a novel strategy for preventing and treating CRA (Takeuchi et al., 2024; Sninsky et al., 2022). Additionally, biomarkers derived from gut microbiota and their metabolites show promise for early screening and risk assessment of CRA (Zhou et al., 2025).

In this study, we conducted metagenomic and metabolomic analyses of fecal samples from CRA patients to identify microbial and metabolic signatures. By integrating multi-omics data, we further explored the role of the gut microbiota in the development of CRA, aiming to provide a scientific basis for the prevention and control of CRC.

## 2 Materials and methods

### 2.1 Study design

All participants were patients or individuals from a physical examination population who underwent colonoscopy at Jiaxing Hospital of Traditional Chinese Medicine, affiliated with Zhejiang University of Traditional Chinese Medicine, between June 2023 and December 2023. This study was approved by the Ethics Review Committee of Jiaxing Hospital of Traditional Chinese Medicine (Ethics No.: JZYLS2024-Y02021) and was conducted in accordance with the Declaration of Helsinki.

The inclusion criteria for patients required a diagnosis of CRA based on both endoscopic and pathological findings, with reference to the Endoscopic tissue sampling - Part 2: Lower gastrointestinal tract. European Society of Gastrointestinal Endoscopy (ESGE) Guideline-2021. Exclusion criteria included: adenomas larger than 3 cm in diameter or requiring surgical resection; a history of hereditary polyps; pathological confirmation or high suspicion of malignancy; active intestinal inflammation; history of colorectal bleeding or surgery; endoscopic procedure within the past 6 months; breastfeeding, current pregnancy, or planning for pregnancy; local residency of less than 3 months; probiotic use within 1 month; and lack of informed consent.

A total of 60 fecal samples were collected from CRA patients and 30 from healthy individuals (all aged between 18 and 70 years). These were assigned to the CRA group and the normal control (NC) group, respectively. All participants completed a questionnaire via a case report form covering demographic, clinical, and lifestyle factors, including age, sex, surgical history, height, weight, dietary habits, medical history, medication use, and tobacco and alcohol consumption. All subjects underwent standard bowel preparation, and colonoscopies were performed by an experienced gastroenterologist.

### 2.2 Sample collection

Fecal samples were collected from eligible participants under fasting conditions (between 5:00 a.m. and 8:00 a.m., prior to colonoscopy prep intervention). Immediately after defecation, samples were placed into 10 mL sterile centrifuge tubes, temporarily stored at −20 °C, and subsequently transferred to a −80 °C freezer for long-term preservation. All samples were processed within 6 months and shipped to Hangzhou Lianchuan Biotechnology Co., Ltd. (Hangzhou, China) for macro-genomic sequencing and metabolomic analysis.

### 2.3 Macro-genomic sequencing

Fecal samples from the CRA group (*n* = 60) and the control group (*n* = 30) were subjected to macro-genomic sequencing. Total genomic DNA was extracted using the EZNA^®^ Stool DNA Kit or the QIAamp DNA Stool Mini Kit. Sequencing was performed on the Illumina HiSeq 2500 platform with a NovaSeq kit. After assessing DNA purity and concentration, libraries were constructed and sequenced.Raw sequencing data were demultiplexed based on barcodes and filtered to remove low-quality reads. Sequence annotation and assembly were conducted using PartekFlow and the MetAMOS pipeline. Non-redundant unigene sets were generated through sequence clustering. Functional annotation was performed using the Kyoto Encyclopedia of Genes and Genomes (KEGG) database via the DIAMOND program. Statistical analysis of KEGG pathways was carried out using one-way ANOVA in the STAMP software package. All data analyses were performed in the R statistical environment. Continuous variables are expressed as mean ± standard error of the mean (SEM) or median with interquartile range, and categorical variables as frequencies. Differences in alpha diversity were assessed using the Wilcoxon test. Beta diversity was evaluated through principal component analysis (PCA) and principal coordinate analysis (PCoA) based on weighted UniFrac distances. Microbial abundance differences were analyzed with the Wilcoxon rank-sum test in STAMP. Statistically enriched taxa (LDA score > 3, Bonferroni-adjusted *p* < 0.05) were identified using LEfSe. Data visualization at phylum and genus levels—including bubble plots, heatmaps, and Sankey diagrams—was conducted with the R packages Rpheatmap, stats, ggplot2, and ggalluvial.

### 2.4 Metabolomics analysis

First, metabolites were extracted from the samples using an organic reagent-based metabolite precipitation method, and multiple quality control (QC) samples were prepared simultaneously. The extracted samples were subjected to randomized sequential on-instrument analysis, with QC samples interspersed before, during, and after the experimental samples to serve as technical replicates for assessing the reliability of the experimental method. Mass spectrometry (MS) scanning was performed on the samples in positive and negative ion modes, respectively.

XCMS software was used for peak extraction and quality control, while metabolite identification was conducted using metaX software. The identified metabolites were annotated using common functional databases. Subsequently, the metabolites were subjected to quantitative analysis, sample correlation analysis, and differential analysis; for the differential metabolites, a series of functional analyses were further performed, including KEGG functional enrichment analysis, mutual network analysis, and metabolite correlation analysis.

Each QC sample was a mixture of equal volumes (or equal amounts) of well-prepared study samples, and these QC samples were interspersed throughout the experiment as technical replicates. Pearson's correlation coefficients were calculated between the abundance profiles of each pair of QC samples, and a correlation heatmap was generated. The higher the correlation between samples, the larger the correlation coefficient and the redder the color in the heatmap. By examining the Pearson's correlation coefficients among QC samples, the reproducibility of metabolite detection could be evaluated: better reproducibility of QC samples indicated more stable instrument performance throughout the entire sample detection and analysis process.

XCMS software was used to extract and align ion peaks across different samples, yielding raw abundance data for each metabolite ion in the samples. Additionally, primary and secondary metabolite identification information for these ions was supplemented. The raw abundance data was generally not used directly and required data quality control and cleaning before it could be applied to downstream analyses. For the ions detected via XCMS, the open-source software metaX was first used for primary identification: the first-stage (MS1) m/z values of the substances were matched against databases including HMDB and KEGG. Due to the presence of numerous isomeric metabolites in these databases, the MS1-based identification results often exhibited a phenomenon where one m/z value corresponded to multiple metabolites; furthermore, the reliability of MS1-based (idms1) reference identification was limited.

Subsequently, an established in-house metabolite tandem mass spectrometry (MS/MS) library was used to match against the MS/MS data of the samples, resulting in metabolite identification results with higher confidence. These results are recommended to be referred to as MS2-based (idms2) identification results. It is important to note that current metabolite databases do not distinguish between species; therefore, the identification results may include matches to metabolites not actually present in the samples. During subsequent data mining, metabolite details can be re-queried using commonly used databases, including HMDB (https://hmdb.ca/), KEGG (https://www.kegg.jp/), and PubChem (https://pubchem.ncbi.nlm.nih.gov/).

XCMS software was used to extract and align ion peaks across different samples, obtaining raw abundance data for each metabolite ion in the samples. metaX software was then employed for data quality control and processing, following these steps:

Removal of low-quality peaks (defined as peaks with >50% missing values in QC samples or >80% missing values in actual samples);

Data normalization using the median normalization method;

Imputation of missing values using the minimum imputation method.

Metabolite identification was conducted using HMDB (Human Metabolome Database, v5.0) and METLIN (v2023), with the following matching criteria: precursor ion mass tolerance ≤ 10 ppm, MS/MS spectral similarity (Dot Product) ≥ 0.8, and retention time deviation ≤ 0.2 min. Mantel tests were performed using Bray-Curtis distance (for microbiome data) and Euclidean distance (for metabolome data), with 999 permutations. For Procrustes analysis, the same distance matrices were used to align the principal coordinate analysis (PCoA) coordinates of microbiome and metabolome data, and significance was evaluated via 1,000 permutation tests.

### 2.5 Statistical analysis

All pairwise comparisons were performed using the two-tailed Wilcoxon rank-sum test. Multiple-group comparisons were conducted using the Kruskal-Wallis H-test. Fisher's exact test was used for analyzing categorical variables. Between-group differences in metabolite profiles (assessed via Euclidean distance) and bacterial communities (assessed via Bray-Curtis distance) were tested.

All statistical analyses were conducted using R software (v3.6.1), which was used for data preprocessing, statistical analysis, and predictive model construction. To control for false positives arising from multiple hypothesis testing, all statistically derived *p-values* were adjusted using the Benjamini-Hochberg false discovery rate (FDR) correction method. Significantly different OTUs/metabolites were defined as those with an FDR-adjusted *p-value* (q-value) < 0.05.

## 3 Results

### 3.1 Clinical baseline data

Participants in the CRA group were older than those in the NC group (*P* < 0.001). The body mass index (BMI) of the CRA group was higher than that of the NC group (*P* = 0.041). The proportion of male participants was higher in the CRA group (*P* = 0.025). The proportion of participants with a history of hypertension was higher in the CRA group than in the NC group (*P* = 0.001; Table 1).

**Table 1:** Clinical baseline data.

**Baseline data**	**NC group (*n* = 30)**	**CRA group (*n* = 60)**	**Statistic**	***P***
Age (At the age of)	29.63 ± 4.93	53.10 ± 11.34	*t* = −13.65	< 0.001
BMI, M (Q1, Q3)	21.90 (19.13, 24.04)	23.33 (22.04, 25.39)	*Z* = −2.04	0.041
**Gender**, ***n*** **(%)**			*χ^2^* = 5.02	0.025
Female	21 (70.00)	27 (45.00)		
Male	9 (30.00)	33 (55.00)		
**History of hypertension**, ***n*** **(%)**			*χ^2^* = 10.48	0.001
No	30 (100.00)	43 (71.67)		
Yes	0 (0.00)	17 (28.33)		
**History of diabetes mellitus**, ***n*** **(%)**			*χ^2^* = 1.81	0.179
No	30 (100.00)	54 (90.00)		
Yes	0 (0.00)	6 (10.00)		
**History of coronary heart disease**, ***n*** **(%)**			*χ^2^* = 0.39	0.533
No	30 (100.00)	57 (95.00)		
Yes	0 (0.00)	3 (5.00)		

*P* < 0.05 was statistically significant.

Although significant differences existed between the CRA group and the NC group in terms of age, BMI, sex, and history of hypertension (*P* < 0.05), the microbiota structure (assessed via PERMANOVA, *R*^2^= 0.08, *P* = 0.003) and the abundances of metabolites associated with Bacteroidota (β = 1.2, 95% confidence interval [CI]: 0.8–1.6, *P* = 0.01), Bacillota (β = 1.45, 95% CI: 0.4–1.2, *P* = 0.003), and Pseudomonadota (β =1.2, 95% CI: 0.45–1.53, *P* = 0.024) remained significantly different after adjusting for these confounders, suggesting their independent associations with CRA.

### 3.2 Metagenomic sequencing results

#### 3.2.1 Intergroup differences in gut microbiota between healthy individuals and CRA patients

**Figure 1:**
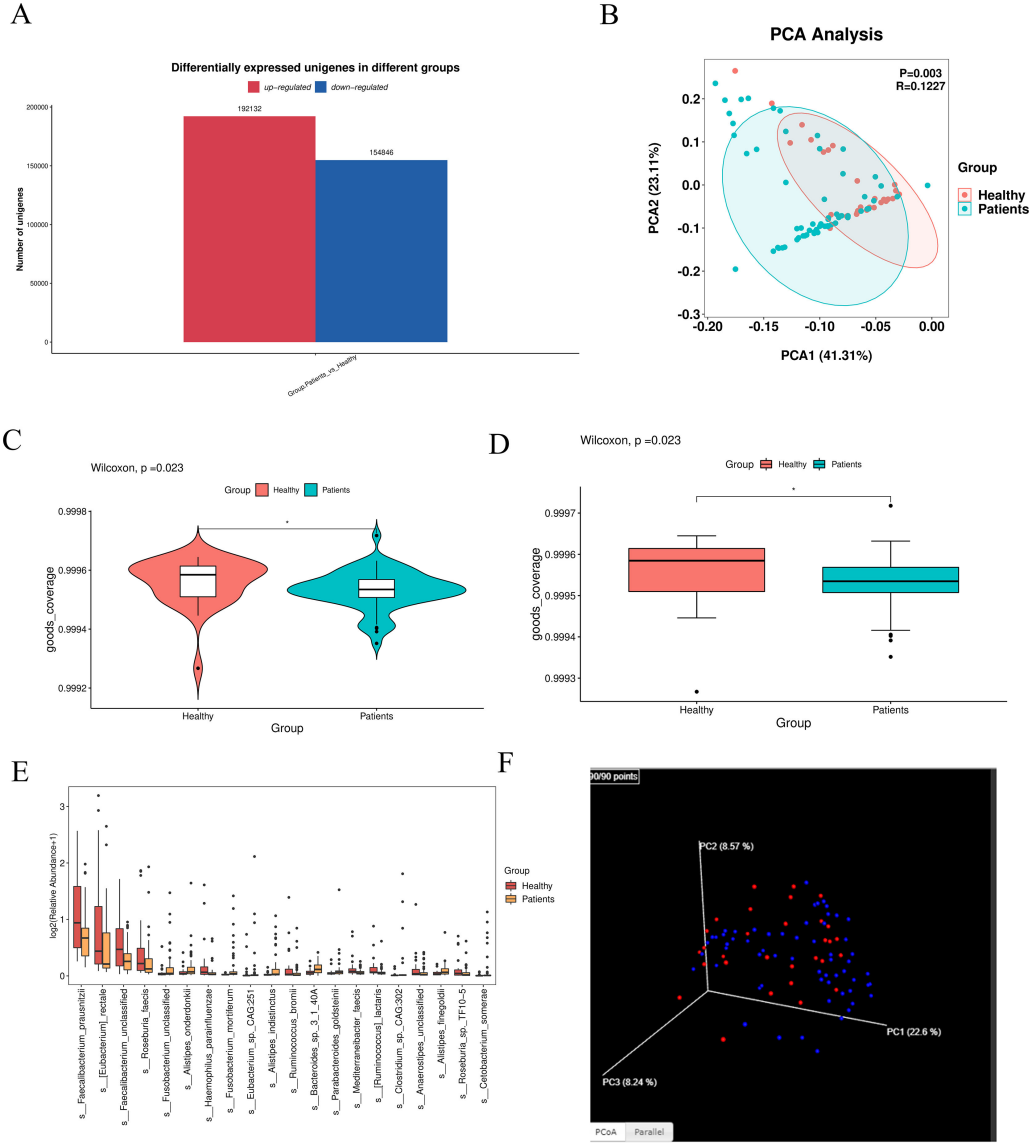
Intergroup differences in gut microbiota between healthy individuals and CRA patients. **(A)** Venn diagram illustrating shared and unique Unigenes between the CRA and NC groups. A total of 2,884,591 Unigenes were identified, with 457,209 unique to the CRA group and 130,644 unique to the NC group. **(B)** PCoA plot showing microbial community similarity and separation between groups. **(C, D)** Violin and box plots from pairwise group comparison, indicating significant differences between groups (** denotes extremely significant difference). **(E)** Box plots of the top 20 most abundant differentially expressed species, displaying abundance distribution across groups. **(F)** 3D PCoA analysis further revealing similarities and partial overlaps, as well as distinct differences, in microbiota composition between CRA patients and healthy individuals.

The bar graph showed the number of shared and unique Unigenes between the CRA group and NC group. In total, 2,884,591 Unigenes were identified across the two groups, with 457,209 and 130,644 unique Unigenes exclusive to the CRA and NC groups, respectively (Figure 1A).

Principal component analysis (PCA) revealed that fecal samples from CRA patients and healthy individuals had similar overall microbiota abundance, with partial overlap in community composition but also distinct floral components (Figures 1B, F). β-diversity analysis was performed via principal coordinate analysis (PCoA) based on Bray-Curtis distance to visualize intergroup differences (Figure 3). The distance between samples was reflected by two principal coordinates (PCo1 and PCo2); samples that clustered closer in the PCoA plot had more similar microbiota compositions. Adonis test results (*p* = 0.01) confirmed significant differences in gut microbiota structure between the two groups.

Difference-in-difference analysis of the two subgroups was visualized using violin plots and box plots, which showed significant intergroup differences (Figures 1C, D); ^**^ denotes highly significant differences (*p* < 0.01). For box plots of divergent species: based on analysis of variance (ANOVA) results, we selected the top 20 most abundant species among those with significant differences (marked as “yes” in the “significance” column) and visualized their abundance across subgroups using box plots (Figure 1E).

#### 3.2.2 Intergroup differences in gut microbiota species abundance

**Figure 2:**
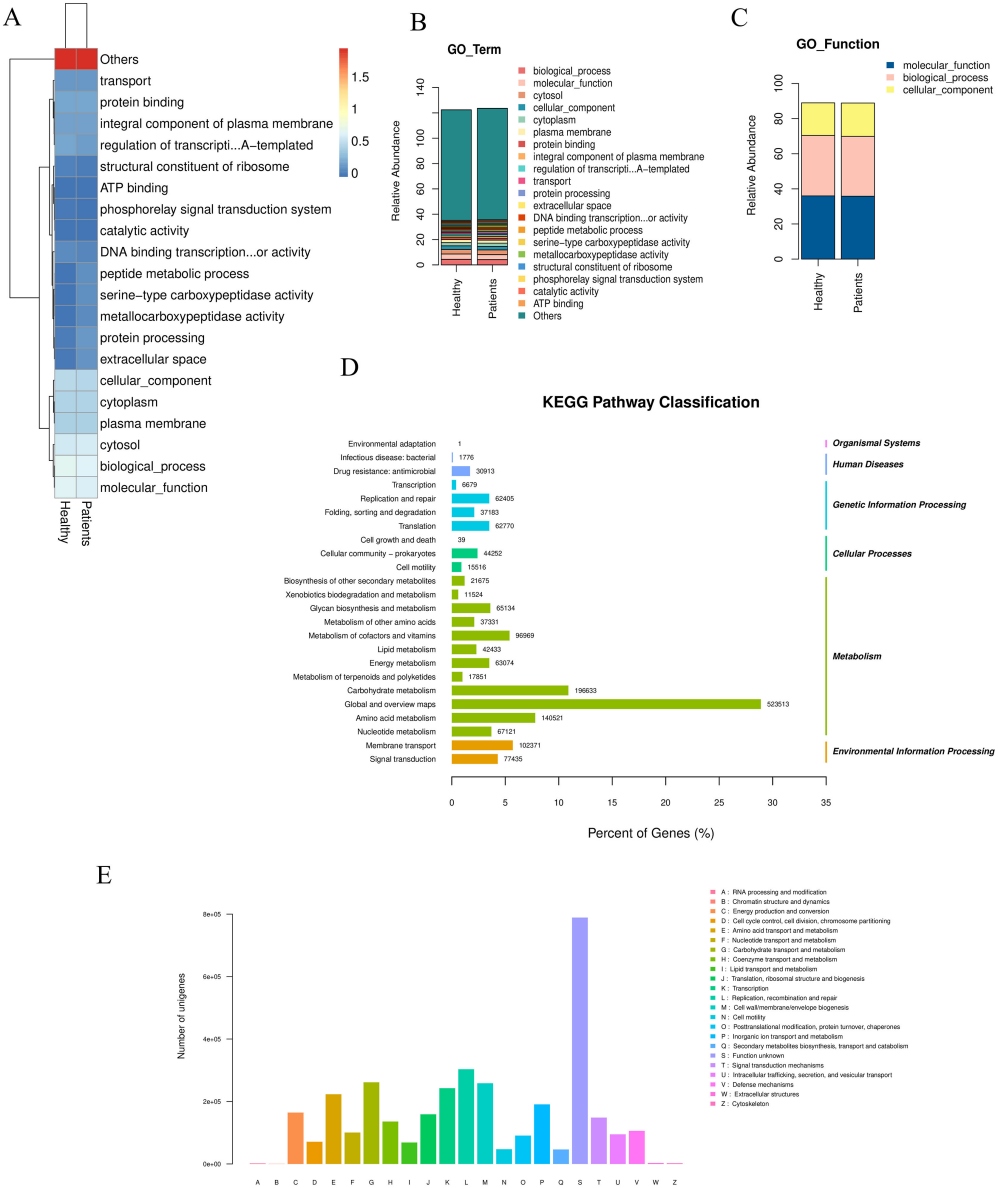
Differences in gut microbiota species abundance between the two groups. **(A)** Clustering heatmap of species abundance at the phylum level. **(B)** Stacked bar chart of species abundance composition at the phylum level. **(C)** Abundance clustering across classification levels based on the GO database. **(D)** Significantly enriched pathways among differentially expressed genes: the top 20 most abundant bacterial species were selected for statistical analysis. The left y-axis indicates the secondary classification of KEGG PATHWAY, the right y-axis shows the primary classification, and the x-axis represents the percentage of Unigenes annotated to each secondary category. Column values indicate the number of Unigenes annotated per category. Results reveal that within Metabolism, the “Global and overview maps” metabolic pathway contains 523,513 differentially expressed Unigenes. **(E)** eggNOG classification statistics: the x-axis represents COG functional categories, the y-axis shows the number of Unigene annotations per category. The legend indicates that the majority belong to the “Function unknown” category.

Based on the statistical table of species abundance at each taxonomic level, the top 20 most abundant species were selected by default (all other species were categorized as “Others”). The abundance of these species in each sample or subgroup was visualized using: Heatmap (species abundance heatmap) (Figure 2A), Stacked bar plot 2b (species abundance at the phylum level) (Figure 2B), Cluster plot 2c (species clustering) (Figure 2C). Stacked bar plots of species abundance at the phylum level (Figure 2B) showed that *Bacteroidota, Bacillota, and Pseudomonadota* were the dominant bacterial phyla in the gut. Heatmap 2a (phylum-level abundance) revealed that, compared to the NC group, the CRA group had a significant increase in the abundance of *Mycoplasmatota, Verrucomicrobiota, Chlamydiota, and Lentisphaerota*, as well as a significant decrease in *Actinomycetota* abundance.At the species level, the abundance heatmap (Figure 2B) showed that the CRA group had a significantly higher relative abundance of *Bacteroides fragilis*, and significantly lower relative abundance of *Phocaeicola plebeius* and *Megamonas funiformis* compared to the NC group. The top 20 most abundant bacteria were subjected to species-level differential statistical analysis (Figure 2D): compared to the NC group, the CRA group exhibited significantly decreased relative abundance of *Faecalibacterium prausnitzii, Eubacterium rectale*, and *Roseburia faecis*, while the relative abundance of *Fusobacterium mortiferum* and *Alistipes spp*. (e.g., *Alistipes* putredinis; specify species if available) was significantly higher.

#### 3.2.3 KEGG enrichment analysis of differential unigenes

In organisms, different genes coordinate to perform biological functions, and pathway-based analysis helps further elucidate the biological roles of these genes. KEGG pathway enrichment analysis (Figure 2D) showed that 523,513 differential Unigenes were enriched in “Global and overview maps” metabolic pathways within the Metabolism category. Description of eggNOG classification statistics (Figure 2E): the x-axis represents each COG functional category, and the y-axis represents the number of differential Unigenes (*n* = 523,513) annotated to each COG category; the legend provides detailed descriptions for each COG functional category. Annotation results (Figure 2E) showed that the largest proportion of Unigenes was annotated to “Function unknown.” Functional annotation of differential Unigenes using the KEGG database, combined with clustering of genes performing the same function, revealed that more genes were enriched in metabolic pathways (primarily within the Metabolism category). This indicated that the gut microbiota play important roles in these metabolic processes. Further metabolic-level functional enrichment analysis identified significant intergroup differences in the following pathways: Metabolic pathways, Biosynthesis of secondary metabolites, Microbial metabolism in diverse environments, Biosynthesis of amino acids, Biosynthesis of cofactors.

#### 3.2.4 Differential expression and enrichment analysis of unigenes

Differentially expressed Unigenes (DEUs) are the most notable results of metagenomic sequencing, as they fully reflect Unigene expression differences between different treatments or sample groups. Different statistical methods were applied to different subgroups and comparison groups: Fisher's exact test: For comparisons without biological replicates, Mann-Whitney U test: For two-group comparisons with biological replicates, Kruskal-Wallis test: For multiple-group comparisons with biological replicates.

Typically, the default threshold for identifying DEUs was: |log_2_ (fold change)| ≥ 1 and *p* < 0.05 (see Supplementary Table S1).

#### 3.2.5 GO enrichment analysis and species annotation of differential unigenes

**Figure 3:**
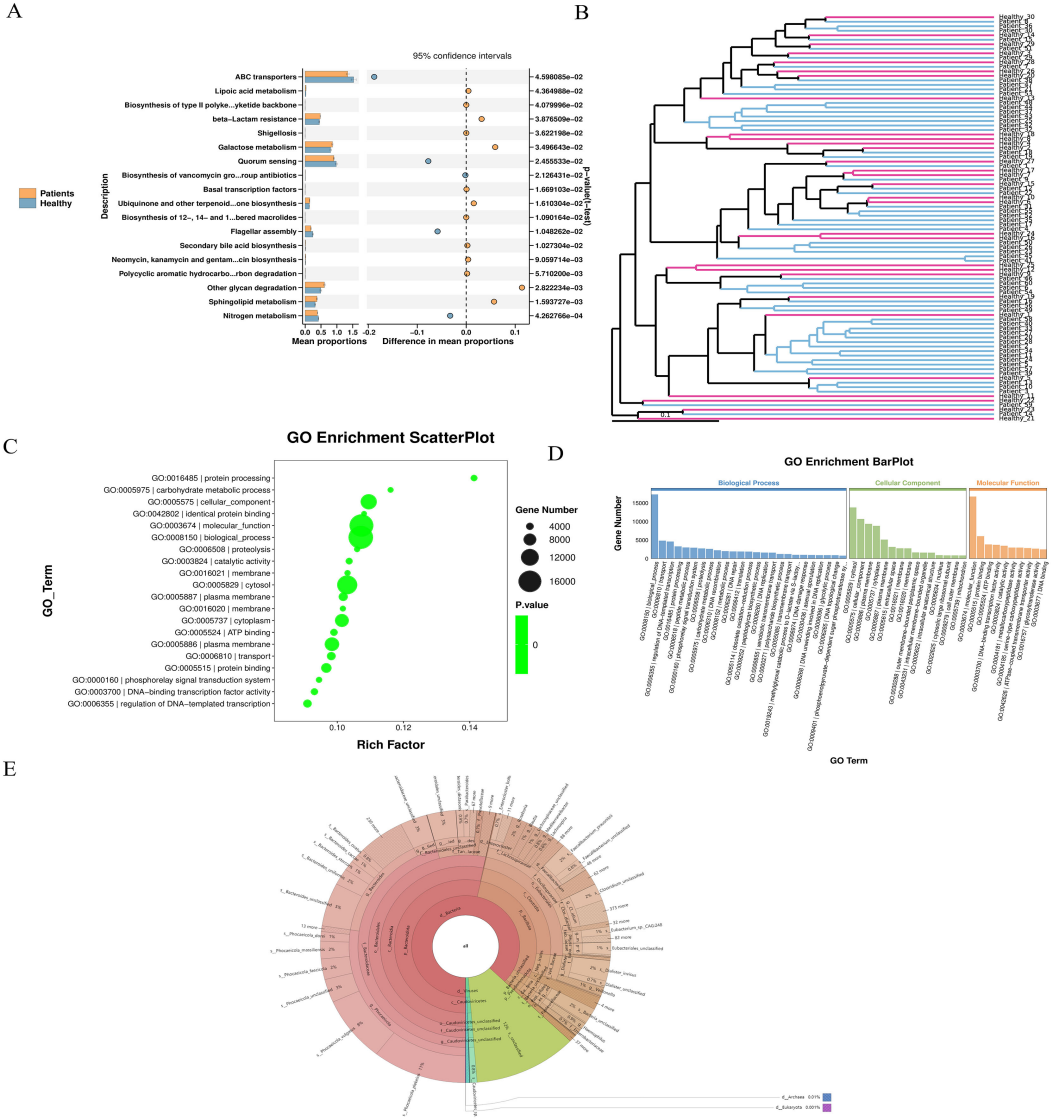
Unigene expression differences and enrichment analysis. **(A)** STAMP plot results indicate a high proportional representation of ABC transporters. **(B)** UPGMA analysis showing sample clustering based on species diversity; a smaller branch length indicates higher similarity between samples. **(C)** GO enrichment bubble chart visualizing enriched GO terms, generated using ggplot2. **(D)** GO enrichment bar chart: bars are colored according to the three major GO categories (Biological Process, Cellular Component, Molecular Function), with same-colored bars representing different GO terms within a category. **(E)** Species abundance and clustering analysis: based on abundance tables, inter-sample clustering was performed using the Bray–Curtis distance matrix, and results were integrated with species abundance profiles for visualization.

GO functional enrichment analysis was conducted by mapping all significantly differentially expressed genes to terms in the Gene Ontology database. The number of genes associated with each term was calculated, and a hypergeometric test was applied to identify GO terms significantly enriched in the differentially expressed gene set compared to the whole genomic background. The STAMP plot results indicated that ABC transporters accounted for the highest proportion among the most significantly different terms (Figure 3A). UPGMA analysis was performed using the Unweighted Pair Group Method with Arithmetic Mean to cluster samples based on a distance matrix. The resulting dissimilarity coefficient between samples was 0.1; a smaller value indicates smaller differences in species diversity between samples (Figure 3B). A GO enrichment bubble chart was generated using ggplot2 to visualize the enrichment results. Among the three major GO categories, biological_process contained the most enriched differential unigenes and the smallest *P-value* (or Q-value) (Figure 3C). The GO enrichment bar chart uses different colors to represent the three major GO categories, with bars of the same color indicating different GO terms within a category. Bar height corresponds to the number of differential unigenes enriched in each term. Specifically, the biological_process category showed the highest enrichment, followed by cellular_component (with the highest enrichment in Cytosol) and molecular_function (Figure 3D). Species annotation was performed using Krona, where circles represent taxonomic levels (from phylum to species) radiating outward. The size of each sector reflects the relative abundance of the corresponding species (Figure 3E). Based on species abundance tables, Bray-Curtis distance matrices were used for inter-sample clustering analysis, integrating both clustering and abundance information.

#### 3.2.6 Advanced analysis

**Figure 4:**
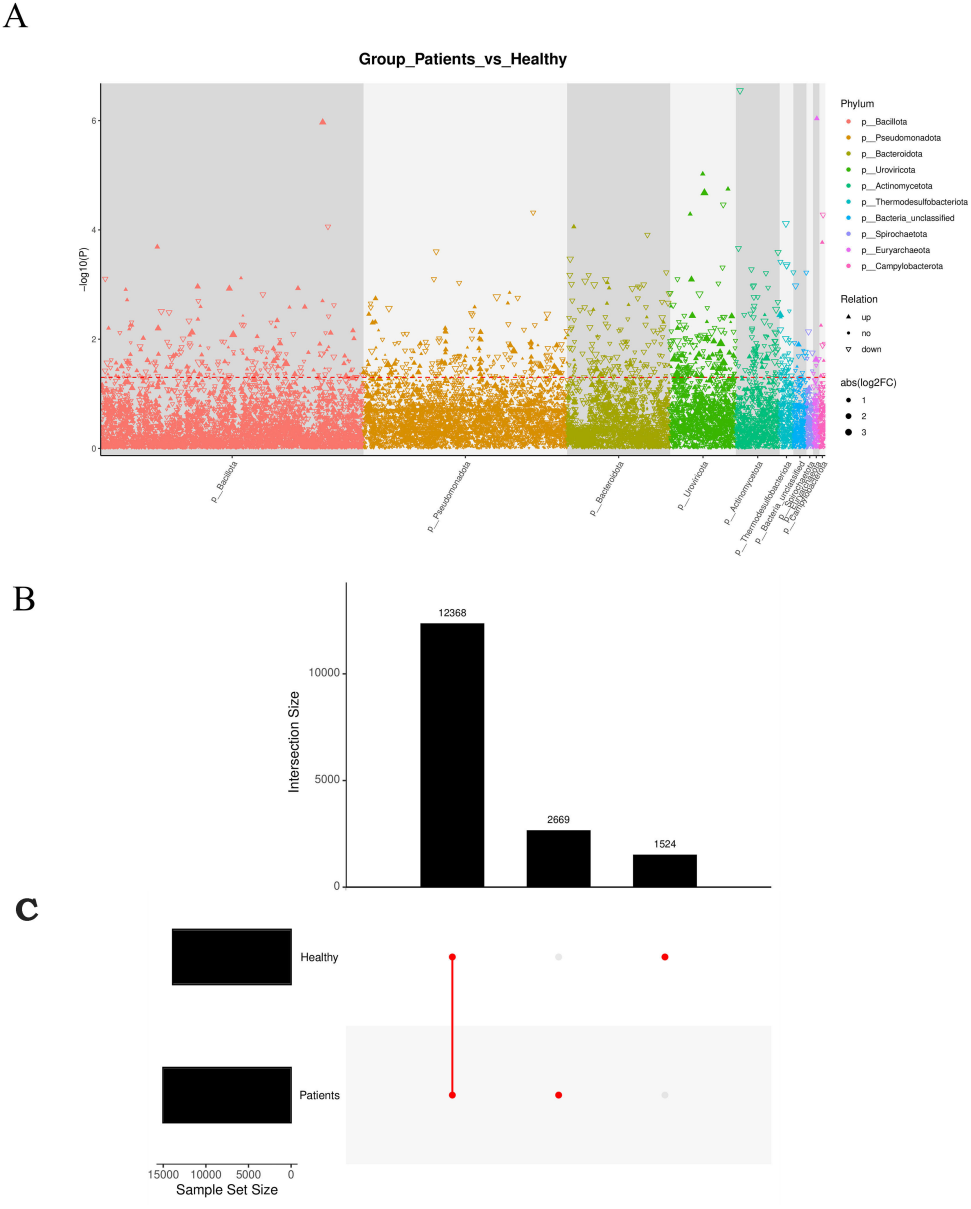
**(A)** Manhattan plot: a type of scatter plot used to display a large number of non-zero, widely fluctuating values—first applied in genome-wide association studies (GWAS) to highlight significantly associated genomic loci. **(B)** Manhattan diagram: the horizontal axis represents taxonomic units at the species level, arranged by their full taxonomic names (from phylum to species). **(C)** Redundancy Analysis (RDA) biplot: each point represents a sample. Closer distances between points indicate higher similarity in community structure. Arrows represent different environmental factors. An acute angle between two factors indicates a positive correlation; an obtuse angle indicates a negative correlation. The length of an arrow reflects the strength of the factor's influence. The projection of a sample point onto an arrow approximates the value of that factor in the corresponding sample.

We identified significantly up-regulated seed-level taxonomic units for each subgroup and higher-level taxa with notable up-regulation. Results are visualized in a Manhattan plot, which revealed that phyla such as Bacillota and Pseudomonadota were prominently up-regulated and contained more genes, whereas Bacteroidota and Uroviricota were associated with more down-regulated genes (Figure 4A). Upset analysis illustrated the overlap between healthy patients and the rest of the sample set. This highlights the importance of focusing on high-discrepancy samples to improve diagnostic specificity in medical research, while also alerting to potential misdiagnosis risks in samples with high overlap (Figure 4B).

RDA analysis, which is a constrained form of principal component analysis (PCA), was performed incorporating the top 10 most abundant taxa along with environmental or clinical indicators measured in each sample (Figure 4C). The results indicated that clinical indicators such as CO and DO had a strong influence on microbial community composition.

### 3.3 Metabolomics results

#### 3.3.1 Metabolite identification

**Figure 5:**
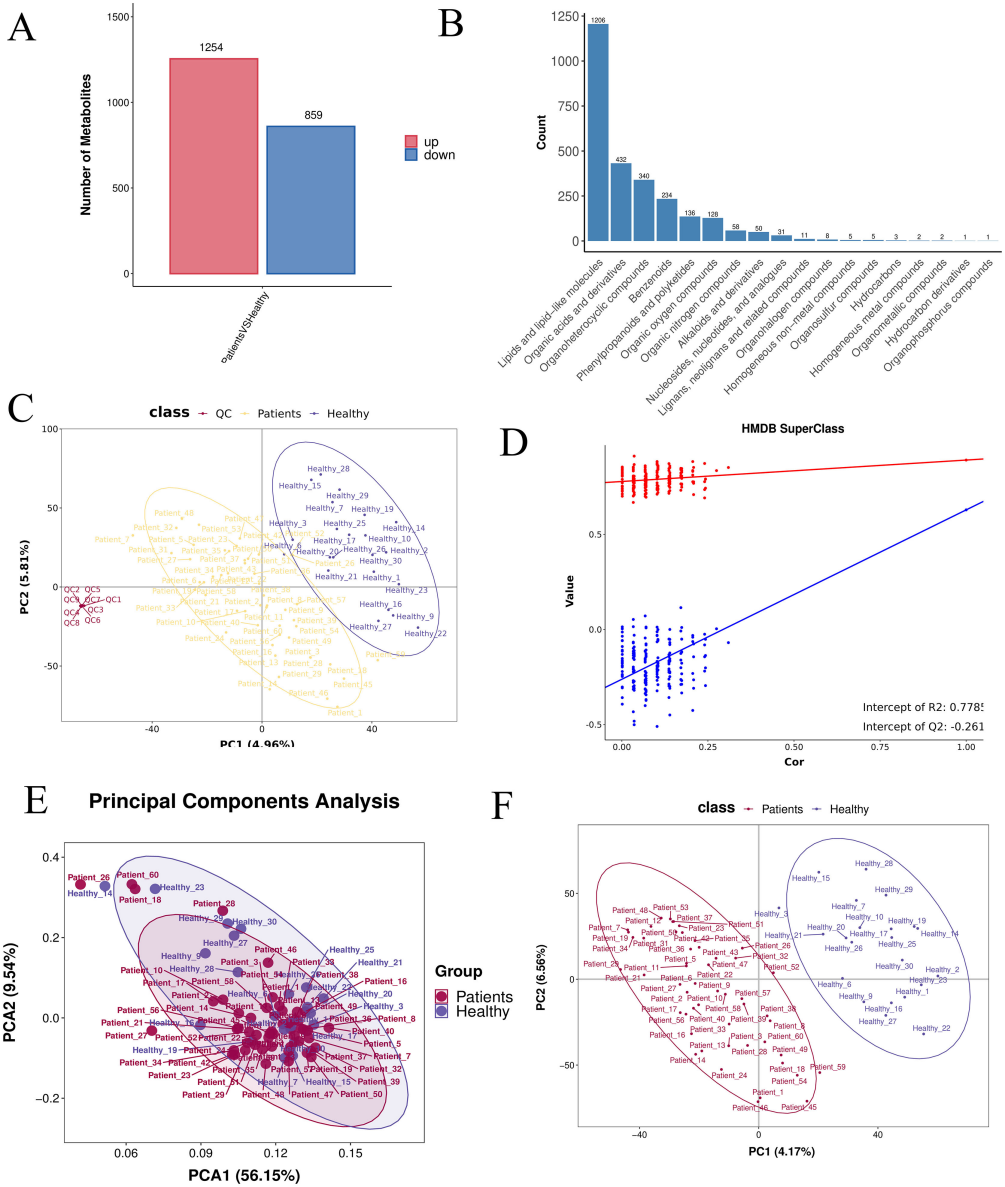
**(A)** Differential ion statistics: the number of significantly up- (red) and down-regulated (blue) ions for each comparison group, based on post-treatment quantitative ion data. **(B)** HMDB SuperClass classification and annotation statistics. **(C)** PLS-DA score plot: the horizontal and vertical axes represent the first (PC1) and second (PC2) principal components, respectively. **(D)** Permutation test plot: after randomly permuting sample group labels, modeling and prediction were repeated; each model corresponds to a set of R^2^ and Q^2^ values. **(E)** PCA analysis between groups. **(F)** PLS-DA analysis between groups.

A differential metabolic ion summary plot, based on quantitative ion data, displayed 1,254 significantly up-regulated and 859 significantly down-regulated ions in the comparative analysis (Figure 5A). Classification and annotation of identified metabolites were performed using the HMDB SuperClass system. Metabolites were cross-referenced with both HMDB and KEGG databases, and the annotated classification results are presented in a bar plot. The horizontal axis represents the number of metabolites, and the vertical axis indicates the SuperClass categories according to HMDB annotation (Figure 5B). The PLS-DA score plot (Figure 5C), permutation test plot (Figure 5D), and principal component analysis (PCA) plot (Figure 5E) collectively confirmed that the model was not overfitted. Pearson's correlation analysis based on abundance values from quality control (QC) samples showed strong correlations within the CRA group and among QC samples, indicating high reproducibility and instrument stability throughout the detection and analytical process (Figure 5F).

#### 3.3.2 Analysis of metabolite differences

**Figure 6:**
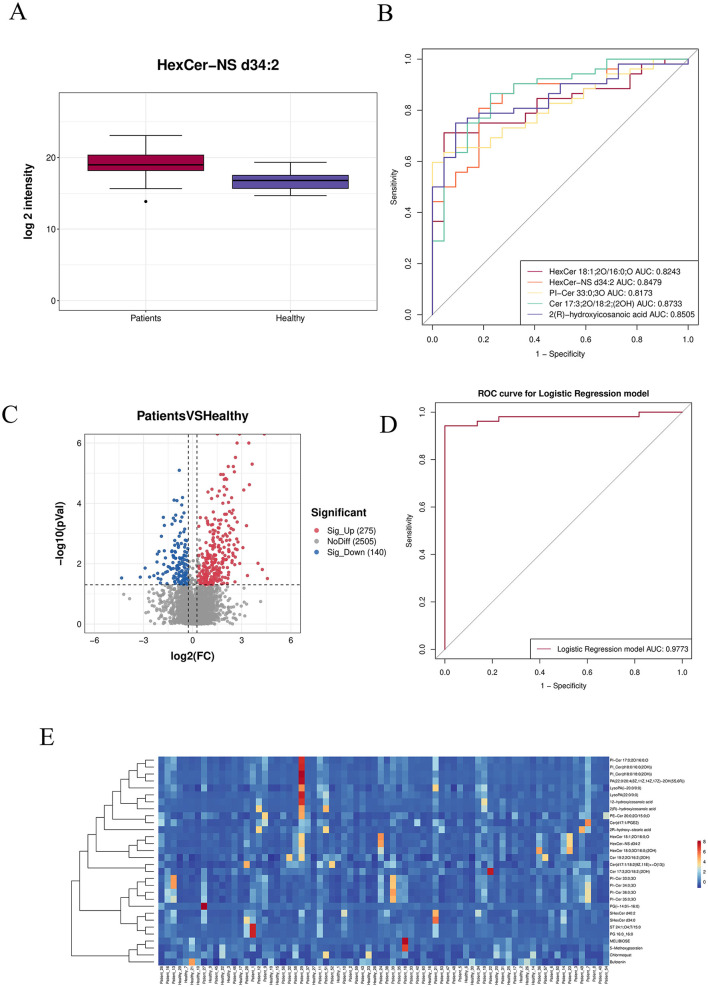
**(A)** Box plot of the top 30 most significantly differential metabolites (smallest *p-values*). **(B)** ROC curves of the top differentially abundant metabolites. **(C)** Volcano plot: red indicates significantly up-regulated metabolites, blue represents significantly down-regulated metabolites, and gray denotes non-significant metabolites. **(D)** Combined ROC curve. **(E)** Heatmap of differential metabolites: the horizontal axis corresponds to samples, and the vertical axis shows selected differentially expressed metabolites.

A box plot of the top 30 most significantly differential metabolites (smallest *p-values*) was generated to illustrate data distribution across groups (see Supplementary Table S2).The horizontal axis represents sample groups, and the vertical axis shows log_2_-transformed abundance values. Significance *p-values* were calculated based on transformed quantitative values (Figure 6A).The top five most significant metabolites (lowest *p-values*) were selected for individual receiver operating characteristic (ROC) curve analysis. A combined ROC curve was also generated using logistic regression modeling. The aggregate AUC reached 0.9773, indicating high diagnostic accuracy (Figures 6B, D). A volcano plot was used to visualize the overall distribution of differential metabolites. The horizontal axis represents log_2_ (fold change), and the vertical axis shows –log_10_ (*p-value*). Significantly up-regulated metabolites are marked in red, down-regulated in blue, and non-significant metabolites in gray (Figure 6C). A heatmap of differential metabolites is presented with samples on the horizontal axis and metabolites on the vertical axis (Top 30 shown by default). Colors indicate relative abundance: red denotes higher abundance, blue lower abundance. Note that Z-score normalization was applied, enabling comparison of the same metabolite across samples horizontally, but not across different metabolites vertically (Figure 6E).

#### 3.3.3 Metabolite enrichment analysis

**Figure 7:**
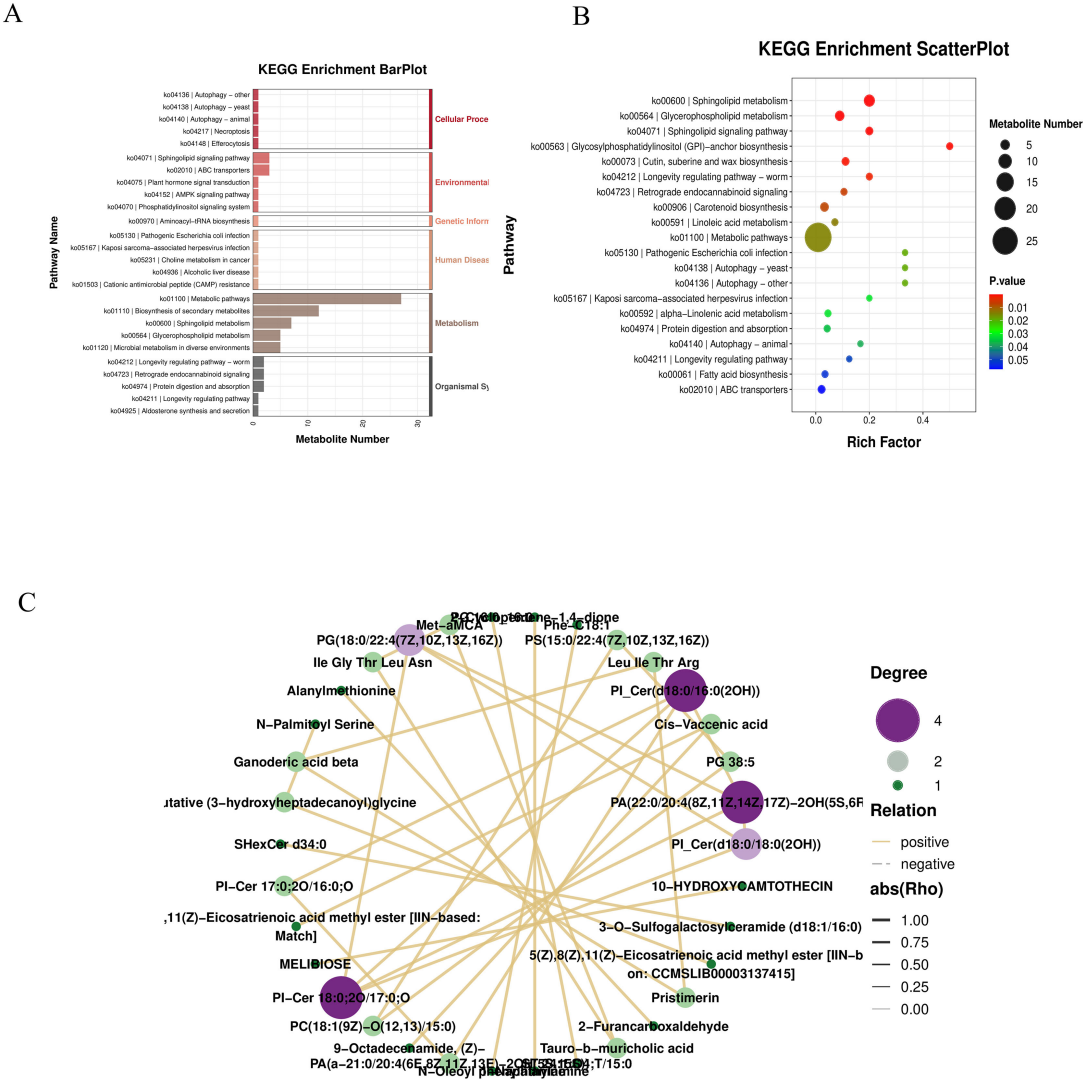
**(A)** KEGG hierarchy bar chart: the horizontal axis indicates the number of differentially abundant metabolites within each pathway, the vertical axis shows pathway names, and the color represents the top-level KEGG category. **(B)** KEGG enrichment bubble plot. **(C)** Correlation network diagram: the top 30 most significant metabolites (lowest *P-values*) were selected. Correlation pairs with |ρ| > 0.7 were identified and used to construct a correlation network. Unlike regulatory network diagrams based on KEGG pathways, this visualization reflects associations between metabolite abundances, highlighting strongly correlated metabolite pairs and emphasizing metabolites of particular interest. Each node represents a metabolite, and nodes with more connections suggest broader functional interactions and potential influence on other metabolites.

KEGG hierarchical clustering bar plots and KEGG enrichment bubble charts indicated that metabolic pathways were the most significantly enriched category (Figures 7A, B). A correlation network diagram was constructed using the top 30 most significantly differential metabolites. Correlation pairs were identified based on computed correlation coefficients, and those with |rho| > 0.7 were selected for network visualization. This network illustrates relationships between metabolite abundances, highlighting strongly correlated metabolites and emphasizing those of particular interest. Each node represents a metabolite, and nodes with more connections indicate metabolites that may influence or be influenced by a larger number of other compounds (Figure 7C).

**Figure 8:**
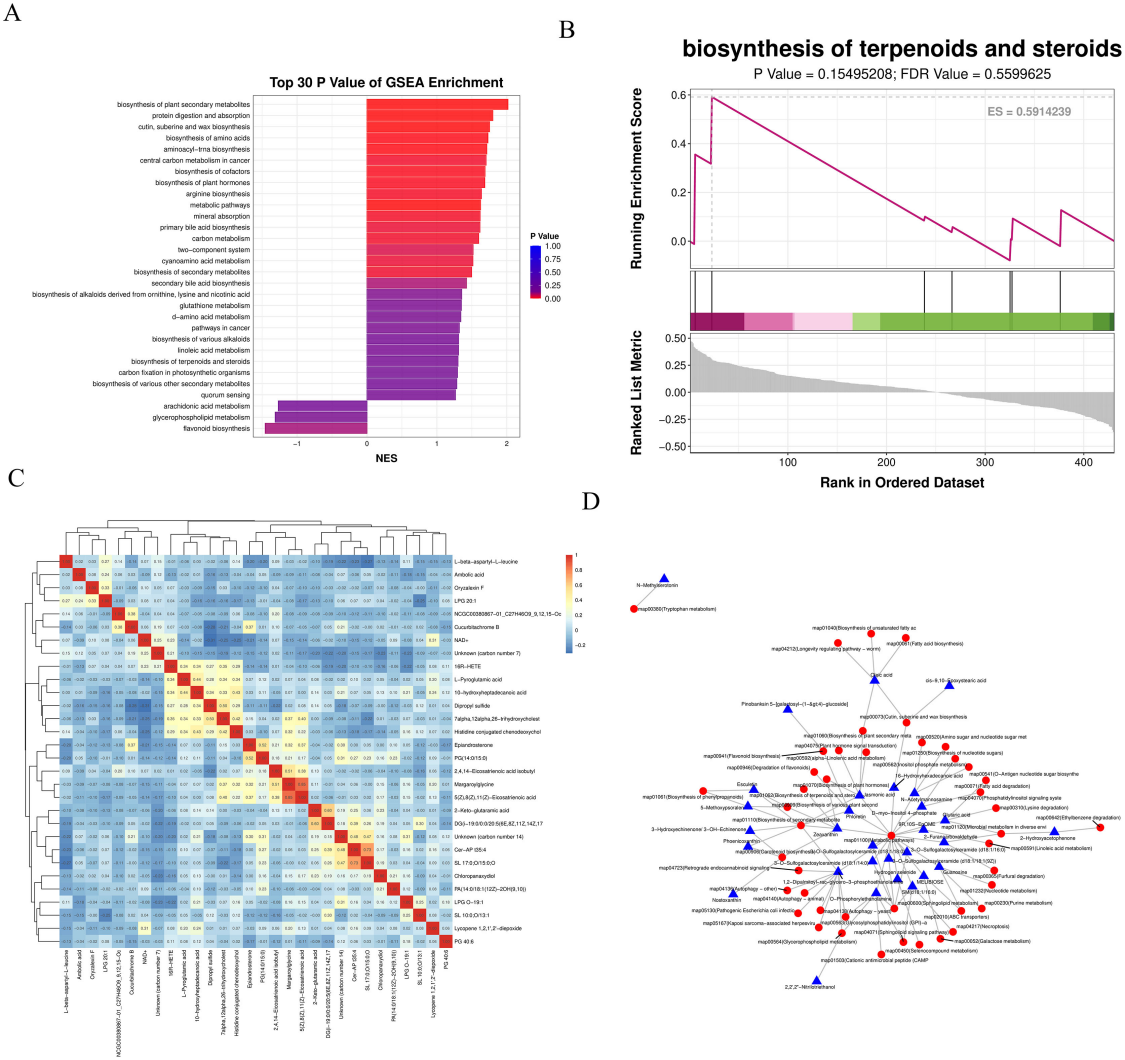
**(A)** KEGG enrichment bar chart: displays the top 30 metabolite sets with the smallest *P-values* and FDR values from GSEA analysis of KEGG pathways. The horizontal axis represents the normalized enrichment score (NES) of the metabolite set, and the vertical axis shows the names of the KEGG metabolite sets. Color indicates the *P-value*. **(B)** KEGG enrichment ES line chart. **(C)** Correlation heatmap of differential metabolites: the top 30 most significant metabolites (lowest *P-values*) were selected. Red indicates stronger positive correlations, and blue indicates stronger negative correlations. The significance of each correlation is assessed based on the *P-value*. **(D)** Regulatory network diagram: depicts interactions between differential metabolites and pathways based on KEGG pathway annotations.

The KEGG enrichment bar graph, based on GSEA analysis of KEGG pathways using the smallest *p-values* and FDR values, revealed that the top 30 metabolites were primarily associated with the biosynthesis of plant secondary metabolites (Figure 8A). The KEGG enrichment ES (enrichment score) line plot currently did not show significant enrichment for terpenoid and steroid biosynthesis pathways (non-significant *p-value* and FDR); however, the ES values suggest a potential weak trend toward enrichment (Figure 8B).

A correlation heatmap was generated using the top 30 most significant differential metabolites. Red indicates a stronger positive correlation, blue a stronger negative correlation. The statistical significance of each correlation was assessed, with smaller *p-values* indicating more reliable correlations (Figure 8C). Based on KEGG annotations of the differential metabolites, a regulatory network between metabolites and pathways was constructed, demonstrating that metabolic pathways form the core of the regulatory mechanism (Figure 8D).

### 3.4 Results of joint analysis between differential species and differential metabolites

Correlation analyses were performed for differential species and differential metabolites in single-cohort and two-cohort comparisons, and correlation graphs were displayed for relationship pairs that met the correlation threshold (threshold: rho ≥ 0.5 and *p* < 0.05); in correlation analyses of differential species and differential metabolites in single-cohort comparisons, if the number of relationship pairs satisfying the threshold was greater than 20, only the 20 pairs with the smallest *p-values* were displayed, and in correlation analyses of differential species and differential metabolites (across all comparisons), if the number of relationship pairs satisfying the threshold was greater than 50, only the 50 pairs with the smallest *p-values* were displayed.

#### 3.4.1 Correlation analysis between differential species

**Figure 9:**
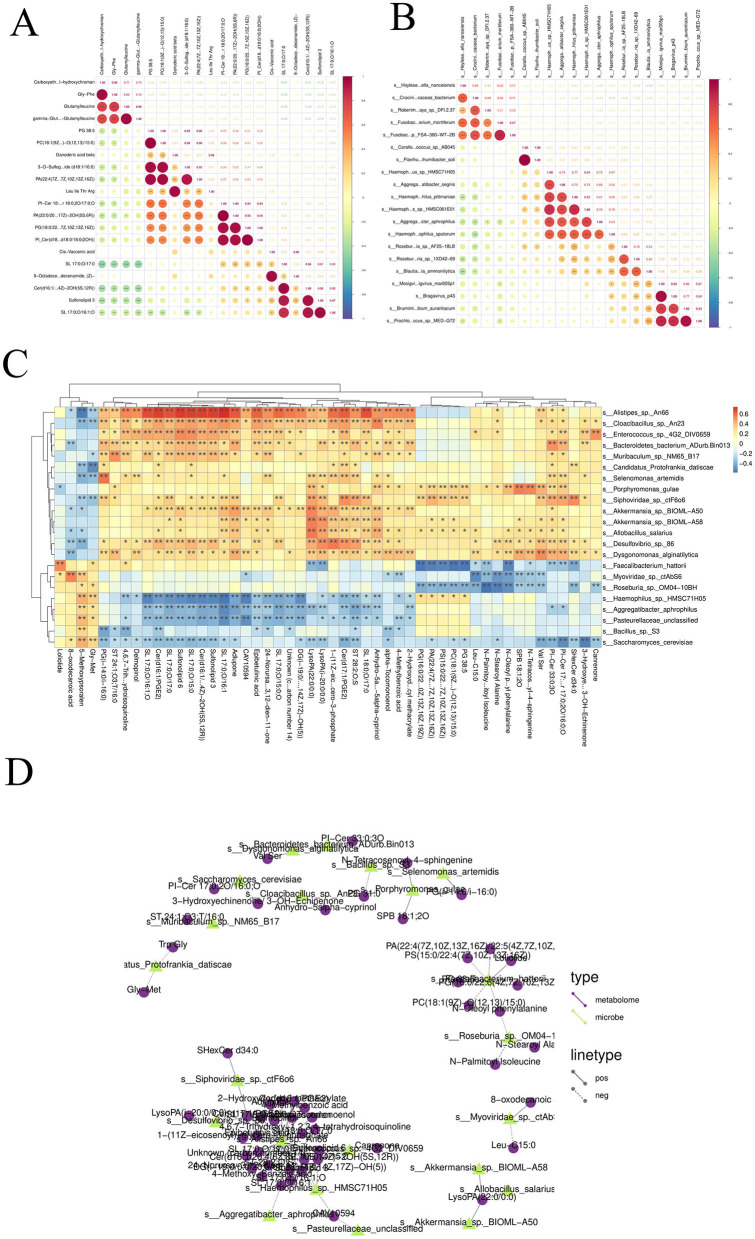
**(A)** Correlation analysis among different species: Spearman correlation coefficients were computed among various species, and a correlation heatmap was generated based on the results. **(B)** Correlation analysis among differential metabolites: Spearman correlations were calculated between differentially abundant metabolites, and a corresponding correlation heatmap was constructed. **(C)** Correlation analysis between differential species and differential metabolites: Spearman correlation analysis was conducted between differential microbial species and differential metabolites. Correlation heatmaps and network diagrams were generated to visualize the associations. **(D)** Correlation network diagram between differential species and differential metabolites.

Spearman correlations were calculated between differential species, and a correlation heatmap was drawn based on the correlation results. Taking the Patients vs. Healthy group comparison as an example: the colors in the graph represent the correlation, with red indicating a positive correlation and blue indicating a negative correlation; the darker the color, the stronger the correlation; the upper right corner represents the value of the correlation coefficient, and the larger the absolute value, the stronger the correlation; the lower left corner represents statistical significance, and significant correlations are marked with asterisks (^*^p < 0.05, ^**^p < 0.01, ^**^p < 0.001). From the above figure, we can identify the significantly correlated differential species (Figure 9A).

#### 3.4.2 Correlation analysis between differential metabolites

Spearman correlations were calculated between differential metabolites, and a correlation heatmap was drawn based on the correlation results. Taking the Patients vs. Healthy group comparison as an example: the colors in the graph represent the correlation, with red indicating a positive correlation and blue indicating a negative correlation; the darker the color, the stronger the correlation; the upper right corner represents the value of the correlation coefficient, and the larger the absolute value, the stronger the correlation; the lower left corner represents statistical significance, and significant correlations are marked with asterisks (*p* < 0.05, ^**^p < 0.01, ^**^p < 0.001). From the above figure, we can identify the significantly correlated differential metabolites (Figure 9B).

#### 3.4.3 Correlation analysis between differential species and differential metabolites

Spearman correlations were calculated between differential species and differential metabolites, and correlation heatmaps and correlation network diagrams were drawn based on the correlation results. Taking the Patients vs. Healthy group comparison as an example: the colors in the correlation heatmap represent the correlation, with red indicating a positive correlation and blue indicating a negative correlation; the darker the color, the stronger the correlation; filled cells represent statistical significance, and significant correlations are labeled with asterisks (*p* < 0.05, ^**^p < 0.01; Figure 9C). For the correlation network diagram between differential species and differential metabolites: taking the Patients vs. Healthy subgroup as an example, different nodes in the diagram represent different microbial flora or metabolites, where microbial flora are represented by triangular nodes (green) and metabolites by circular nodes (purple); a line between a microbial flora and a metabolite represents a significant correlation between the two, with a solid line indicating a positive correlation and a dashed line indicating a negative correlation. Note: only relationship pairs that satisfy the set correlation threshold and have *p* < 0.05 are displayed in the results (Figure 9D).

#### 3.4.4 Mantel test analysis

**Figure 10:**
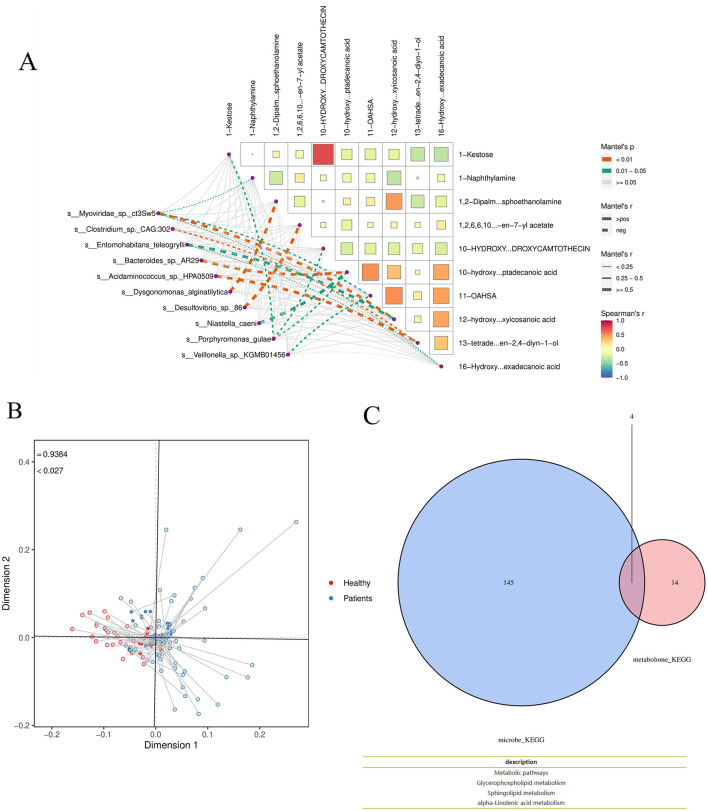
**(A)** Mantel test Analysis **(B)** Procrustes analysis T **(C)** Functional analysis. Taking the Patients_vs_Healthy group as an example, Venn analysis was conducted on the functional items of the two omics differences.

Using the Patients_vs_Healthy grouping as an example, a Mantel test was performed to analyze the correlation between differential microbial species and differential metabolites. In the corresponding heatmap, the color of each block indicates the strength of the correlation between metabolic groups: redder shades represent correlation coefficients closer to 1, indicating a stronger positive correlation, while bluer shades represent coefficients closer to −1, indicating a stronger negative correlation. The network diagram in the lower left corner shows the correlation results between the top 10 most significant differential species and the top 10 most significant differential metabolites. Node color represents the *p-value*, and line thickness corresponds to the r-value, with thicker lines indicating stronger correlations. For clarity of visualization, the 10 species and metabolites with the smallest *p-values* are selected by default. The species and metabolite datasets can be interchanged during analysis to obtain correlation results among the species themselves and between species and metabolites overall. The Mantel test analysis and visualization were conducted using the Correlation Network Heat Map Cloud tool. If available, environmental or clinical metrics, as well as alpha diversity indices, can also be incorporated into the analysis (Figure 10A).

#### 3.4.5 Procrustes analysis

Procrustes analysis was applied to differential species and metabolites using the Patients_vs_Healthy subgroup as an example. In the resulting figure, different colors represent different subgroups; each line segment corresponds to one sample, with one end indicating the microbiome data point and the other end (solid triangle) representing the metabolite data point from the same sample. The connecting line represents the residual vector between the two ordination configurations, with shorter lines indicating higher consistency between the two datasets. The value m^2^ denotes the sum of squared errors between the configurations—a smaller value indicates greater consistency. The *P-value* was generated via Monte Carlo simulation, where *p* < 0.05 indicates a statistically significant agreement between the datasets, and *p* ≥ 0.05 indicates non-significance (Figure 10B).

Functional analysis: Using the Patients_vs_Healthy subgroup as an example, Venn analysis was performed on functional entries derived from the two omics datasets. The results indicate overlapping functional pathways, including Metabolic pathways, Glycerophospholipid metabolism, Sphingolipid metabolism, and Alpha-linolenic acid metabolism (Figure 10C).

## 4 Discussion

In recent years, the impact of gut flora on diseases has received increasing attention (Li et al., 2025; Shen et al., 2024). The intestinal microbiota consists of intestinal microorganisms and the intestinal environment, and is known as the “second human genome” due to its complex composition. This “second human genome” is not only involved in the decomposition, digestion, and absorption of amino acids, sugars, and fats, but also provides a variety of nutrients required by the host and promotes the growth and development of intestinal epithelial cells; in addition, it inhibits the proliferation of pathogenic bacteria, resists pathogen invasion, and regulates the intestinal mucosal immune response (Ghosh et al., 2022). In this study, fecal samples from CRA patients were analyzed using 16S rRNA gene sequencing and untargeted metabolomics. Microbial variations identified in this prospective study distinguished CRA patients from controls, and beta diversity analysis (PCA and PCoA) showed statistically significant differences in microbial clustering between the CRA group and controls (*p* < 0.05). Additionally, taxonomic statistical analyses were performed at the phylum and genus levels to clarify the dominant microbiota composition. At the phylum level, *Bacillota (Thick-walled Bacteria), Bacteroidota, Pseudomonadota, Actinomycetota (Actinobacteria), and Clostridia-related* taxa dominated in terms of relative abundance. Differential analysis of intestinal flora at the species abundance level revealed significant differences in the taxonomic composition of intestinal flora between healthy individuals and CRA patients. KEGG functional enrichment analysis showed that the differential flora were mainly involved in metabolic processes, including metabolic pathways, biosynthesis of secondary metabolites, microbial metabolism in diverse environments, biosynthesis of amino acids, and biosynthesis of cofactors. In this study, three intestinal microbial species—*Fusobacterium mortiferum, Alistipes* spp., and *Bacteroides fragilis*—were validated to distinguish between healthy individuals and CRA patients, among which *Alistipes* spp. showed the best discriminatory ability. *Alistipes*, a relatively new sub-branch of the phylum Bacteroidota, is commonly isolated from the human intestinal microbiota and is known to not only provide energy for intestinal epithelial cells but also regulate the host's immune response (Song et al., 2024). In some CRC patients, the abundance of *Alistipes* in the gut was significantly altered, which may be closely related to the development of CRC (Wada et al., 2022). *Alistipes* may also metabolize and produce certain biologically active substances that are potentially anticarcinogenic; however, some harmful *Alistipes* strains can promote inflammation and tumor formation (Vallianou et al., 2021). *Fusobacterium mortiferum* commonly affects human health as a pathogenic bacterium (Ow et al., 2023): its abundance has been found to be increased in CRA patients with APC mutations, and such enrichment in APC-mutant patients has been shown to be associated with a higher incidence of CRC (Siskova et al., 2020). Experiments on CRC mouse models have shown that *Fusobacterium mortiferum* may be involved in distant metastasis of CRC, and other studies have reported elevated *Fusobacterium mortiferum* abundance, reduced T-cell infiltration, and decreased overall survival in CRC patients (Deng et al., 2025). *Bacteroides fragilis* can induce a series of inflammatory responses via *Bacteroides fragilis* toxin (BFT), which can lead to chronic intestinal inflammation, tissue damage, and ultimately CRC (de Vos et al., 2022). Simpson et al. found that the pro-carcinogenic bacterium *Bacteroides fragilis* has the potential to initiate and promote CRC (Simpson et al., 2021). In conclusion, we hypothesize that *Fusobacterium mortiferum, Alistipes* spp., and *Bacteroides fragilis* are closely associated with CRA and even CRC, and that increased abundance of these taxa may promote CRC progression (Banaszak et al., 2023).

Several metagenomic studies have found that the abundance of Bacteroidota (Bacteroidetes) is reduced in the intestines of patients with colorectal adenomas, whereas the abundance of Pseudomonadota (Proteobacteria) and Bacillota (Thick-walled Firmicutes) is significantly increased (Leeuwendaal et al., 2022). In particular, the enrichment of conditional pathogenic bacteria such as Fusobacterium nucleatum and Escherichia coli is strongly associated with the development of colorectal adenomas (Zhao et al., 2019). These flora may be involved in adenoma formation by promoting inflammatory responses, disrupting intestinal barrier function, and inducing DNA damage. Metabolites of intestinal flora play an important role in the development of colorectal adenomas (Zhang et al., 2023). Short-chain fatty acids (SCFAs, such as butyric acid, propionic acid, and acetic acid)—the main products of dietary fiber fermentation by intestinal flora—have anti-inflammatory properties, maintain intestinal barrier integrity, and inhibit tumor cell proliferation (Ozcam and Lynch, 2024). It has been found that patients with colorectal adenomas have a significantly lower abundance of butyrate-producing bacteria in their intestines, leading to decreased butyric acid levels, which may weaken its protective effect on the intestinal mucosa. In addition, secondary bile acids produced by gut flora metabolism are significantly elevated in patients with colorectal adenomas, and these metabolites may promote adenoma formation by inducing oxidative stress and DNA damage. Interactions between the intestinal flora and the host involve complex molecular mechanisms (Rinninella et al., 2019). Studies have shown that flora metabolites can affect cell proliferation, apoptosis, and differentiation by regulating host cell signaling pathways (e.g., Wnt/β-catenin, NF-κB, and PI3K/AKT pathways) (Liu et al., 2021). For example, Clostridium perfringens promotes abnormal proliferation of colorectal epithelial cells by activating the Wnt/β-catenin signaling pathway (Han et al., 2021). In addition, flora metabolites can influence host gene expression through epigenetic modifications (e.g., DNA methylation and histone modifications), which may be involved in adenoma development (Zolkiewicz et al., 2020).

Metabolomics results showed that among differential metabolite ions, 1,254 were significantly upregulated and 859 were significantly downregulated. Partial least squares discriminant analysis (PLS-DA) score plots, permutation test plots, and principal component analysis (PCA) results indicated that the model was not overfitted. ROC curves of top differential metabolites and the combined ROC curve showed an AUC of 0.9773, indicating high diagnostic value and high accuracy of the differential targets. The differential metabolites mainly included 3-methyl-2-oxindole, histidylproline, nonaethylene glycol, tetrabutylammonium, N-(5-acetamidopentyl)acetamide, trans-ferulic acid, palmitamide (cis), cis-9,12-octadecadien-1-ol, and threonylproline, which are mainly categorized into tryptophan metabolism-related metabolites, protein degradation products, amides, and phenolic acid metabolites. KEGG hierarchical cluster heatmaps and KEGG enrichment bubble plots showed that the top 30 metabolites (ranked by KEGG enrichment significance) were mainly involved in the biosynthesis of plant secondary metabolites; these metabolites can regulate the composition of intestinal flora, promote the growth of probiotics (e.g., Bifidobacterium and Lactobacillus), and inhibit the proliferation of pathogenic bacteria, thereby reducing intestinal inflammation and the production of carcinogens (e.g., secondary bile acids) (Chen et al., 2025). Metabolomics analysis showed that compared to the healthy group, patients with colorectal adenomas had significantly lower levels of short-chain fatty acids (SCFAs, such as butyric acid, propionic acid, and acetic acid) in their intestines, whereas the levels of secondary bile acids (e.g., deoxycholic acid and lithocholic acid) were elevated (Chandrasekaran et al., 2024). Reduced butyric acid levels in the intestines of CRApatients may weaken its protective effect on the intestinal mucosa, while the accumulation of secondary bile acids may promote adenoma formation by inducing oxidative stress and DNA damage (Satchwell et al., 2024). It was further shown that butyric acid not only inhibits tumor cell proliferation but also exerts antitumor effects by regulating histone deacetylase (HDAC) activity to influence gene expression. In addition, this study found that the levels of polyamine metabolites were significantly increased in patients with colorectal adenomas, and these metabolites may be involved in adenoma development by promoting cell proliferation and inhibiting apoptosis (Sasso et al., 2023).

Mantel test analysis revealed that the metabolite 10-Hydroxycamptothecin exhibited the strongest correlation with the microbial species 1-Kestose, while the differential species Bacteroides was most strongly correlated with the metabolite hydroxyoctadecanoic acid. Additionally, Acidaminococcus showed the strongest correlation with the metabolite 13-tetradecen-2, 4-diyn-1-ol. Procrustes analysis, combined with Venn analysis of functional entries derived from the two omics datasets, highlighted the following significantly enriched pathways: Metabolic pathways, Glycerophospholipid metabolism, Sphingolipid metabolism, and Alpha-linolenic acid metabolism. Glycerophospholipid metabolism contributes to CRA development through multiple mechanisms, including inflammation, oxidative stress, and activation of signaling pathways (Sasso et al., 2023). Specifically, phosphatidylinositol—a key glycerophospholipid metabolite—serves as an important regulator of the PI3K/AKT/mTOR pathway, whose aberrant activation may promote cell proliferation and inhibit apoptosis. Additionally, mutations in the APC gene, frequently observed in colorectal adenomas, lead to constitutive activation of the Wnt/β-catenin pathway. Dysregulated lipid metabolism may synergize with this pathway to accelerate tumor progression (Nigam et al., 2022). In sphingolipid metabolism, reduced ceramide levels and elevated sphingosine-1-phosphate (S1P) contribute to inhibited apoptosis and aberrant cell proliferation. S1P helps maintain the tumor microenvironment by recruiting immune cells such as macrophages and promoting the release of proinflammatory factors. Abnormal sphingolipid metabolism may also increase reactive oxygen species (ROS) production, leading to DNA damage in intestinal epithelial cells (Suler Baglama and Trcko, 2022). Growing evidence indicates that gut microbial dysbiosis is a key environmental factor in the development of CRC and its precancerous lesions (Lu et al., 2019). Adenomatous tissues are characterized by increased bacterial abundance and diversity, particularly within genera such as Aspergillus and Clostridium (Yeoh et al., 2021). During CRC development, certain bacteria—including Bacillus and Clostridium—can trigger mucosal inflammation, mediate oncogenic signaling pathways, and suppress host immune responses, thereby promoting adenoma formation (Braga et al., 2024). The presence of adenomas may further disrupt microbial balance, increasing the abundance of opportunistic pathogens such as Pseudomonas and Streptococcus, which alter intestinal homeostasis, enhance inflammatory infiltration, and directly or indirectly elevate adenoma risk.

Fecal analyses of CRA patients and healthy individuals revealed no significant differences in microbial abundance or α-diversity, but β-diversity was markedly altered. These differences were primarily driven by taxa within the phylum Aspergillus and were not significantly associated with patient gender. Many studies have identified Clostridium difficile as an important bacterium associated with CRA. Short-chain fatty acids (SCFAs) play a crucial role in maintaining intestinal mucosal homeostasis. Group 3 innate lymphoid cells (ILC3s) in the colon express numerous SCFA receptors. Genetic deletion of these receptors impairs ILC3 proliferation and IL-22 production, weakening antimicrobial immunity. SCFAs, as major microbial metabolites, can directly activate ILC3s through receptor binding. Tryptophan, a metabolic byproduct of gut microbiota, serves as both a ligand for the aryl hydrocarbon receptor (AHR) and an energy source for lactobacilli. AHR is a key transcriptional regulator supporting ILC3 function and differentiation. Additionally, gut microbiota can directly stimulate ILC3s by inducing IL-1β and IL-23 production.

**Figure 11:**
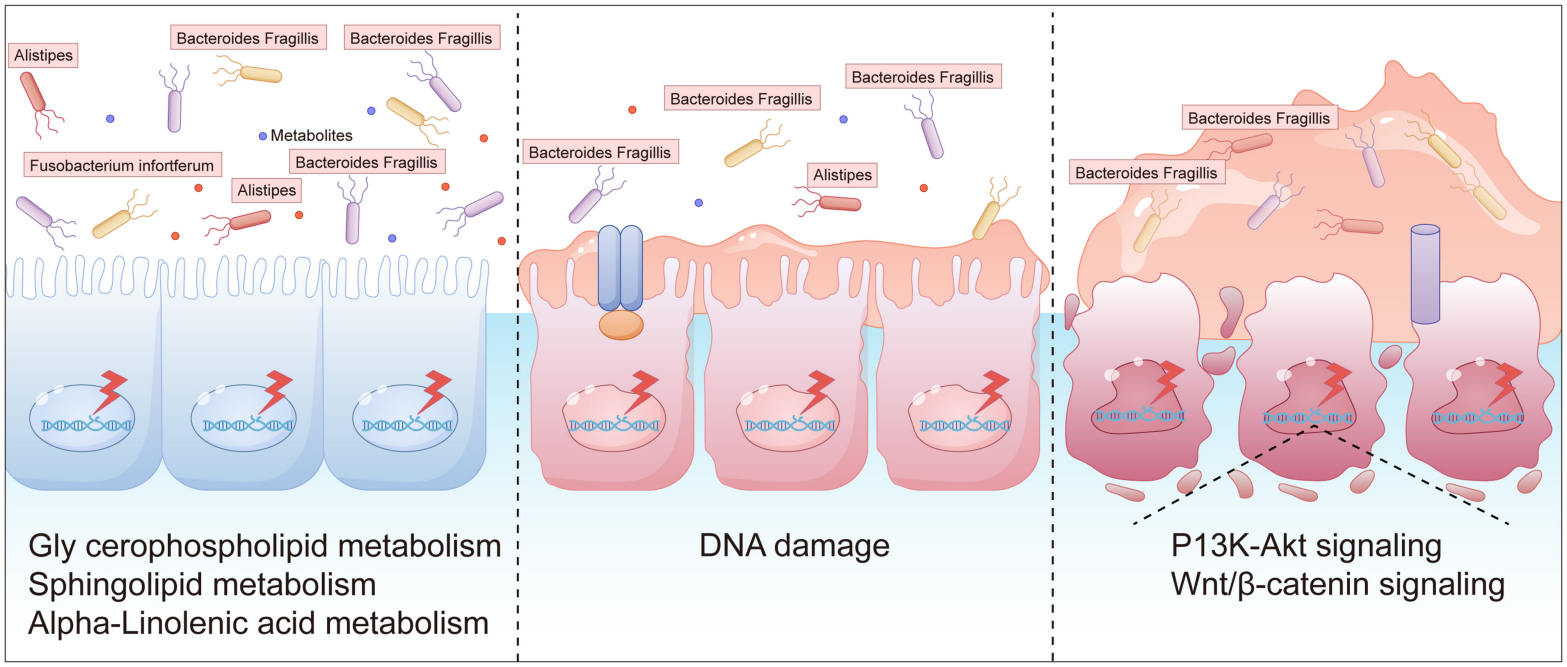
A schematic diagram of intestinal polyps caused by intestinal flora imbalance (The graphical representations were independently designed using AI Illustrator based on data from: (i) inter-group differences in gut microbial species abundance (Section 3.2.2), (ii) differential metabolite analysis (Section 2.3.2), and (iii) Mantel test results.).

Gut dysbiosis is both a cause and consequence of chronic inflammation. Although acute inflammation helps eliminate toxins and initiate tissue repair, persistent overexpression of inflammatory factors or impaired resolution pathways can lead to chronic inflammation, which promotes tumor development through chemotaxis, proliferation, angiogenesis, invasion, and metastasis. In the digestive system, controlling chronic inflammation is critical for preventing and improving the prognosis of colorectal, pancreatic, gastric, and hepatic cancers. Elevated abundances of Clostridium perfringens and Fusobacterium nucleatum have been observed in fecal samples from CRA patients, with progressive increases from healthy individuals to CRA and CRC cases. In conclusion, the abundance of Clostridium perfringens correlates positively with the degree of atypia in polyp tissues. Even in small polyps, Clostridium abundance was higher than in normal mucosa. A schematic diagram summarizing these findings is provided (Figure 11).

Nevertheless, this study is subject to several limitations. A principal limitation is the complexity and heterogeneity inherent in data derived from multi-omics approaches. For example, metagenomic and metabolomic analyses frequently uncover a wide range of microbial and metabolic alterations associated with CRA; however, discerning causative factors from mere associations remains a significant challenge. Furthermore, the interpretation of multi-omics data is often complicated by confounding variables such as age and body mass index (BMI), which can impact both the microbiome and metabolome. Consequently, the results of this study should be interpreted with caution and warrant further experimental validation.

The present study identified a distinct compositional signature that links alterations in gut microbiota and metabolomic profiles to the progression of CRA. However, it is important to note that these correlative findings do not imply causation. To transition from associative mapping to mechanistic validation, future research should employ hypothesis-driven functional frameworks. Specifically, germ-free murine transplantation models could be utilized to isolate the oncogenic potential of candidate bacterial consortia, while vertical microbiota transfer experiments in genetically predisposed models might elucidate temporal pathogenicity. Additionally, *in vitro* 3D organoid co-culture systems—enriched with bacterial metabolites (e.g., the lithocholic acid derivatives identified in this study)—would facilitate a precise examination of microbe-associated molecular pattern (MAMP) signaling dynamics through transcriptomic and phosphoproteomic analyses. These complementary approaches are essential to bridging the current correlative gap by testing whether microbial biomarkers serve as primary initiators in adenoma genesis or function as microenvironmental modulators that promote tumor evolution.

Incorporating diverse fecal metabolomic biomarkers into current CRA screening protocols holds great potential to substantially improve early detection and diagnosis. Initially, fecal metabolomics facilitates the identification of specific metabolites that are altered in patients with CRA relative to healthy individuals. For example, research has demonstrated that certain bioactive lipids—including polyunsaturated fatty acids, secondary bile acids, and sphingolipids—are elevated in individuals with CRA; these metabolites may signify early events in carcinogenesis and serve as potential biomarkers for early detection (Kim et al., 2020). Furthermore, integrating metabolomics with microbiome data can yield a more comprehensive understanding of the gut environment in CRA patients. An integrative analysis of fecal metagenomics and metabolomics has uncovered significant interactions between bacterial and host metabolites, which outperform traditional diagnostic methods [e.g., fecal occult blood test (FOBT)] in CRC diagnosis (Clos-Garcia et al., 2020)—indicating that a similar methodology could be applied to CRA screening. Additionally, targeted metabolomic analyses have identified nucleosides in serum samples that exhibit a strong correlation with CRA and CRC; these findings hold potential for translation to fecal metabolomics, enabling the identification of analogous nucleoside biomarkers in stool samples and thus providing a non-invasive screening tool (Zheng et al., 2023). The development of diagnostic models utilizing metabolomic data represents another promising avenue. For instance, multi-matrix metabolomic analyses have been employed to develop classifiers capable of distinguishing between cancer subtypes, cancer stages, and microsatellite status based on significant metabolites; such models could be adapted to specifically target adenomas, potentially enhancing early detection rates (Zhang et al., 2024). Moreover, fecal fatty acid profiling has demonstrated potential as a CRC screening tool, with alterations in the fecal fatty acid metabolome observed in CRC patients compared to healthy controls; this methodology could be extended to adenoma screening by identifying specific fatty acids indicative of adenoma presence (Zhang et al., 2021). Finally, incorporating these metabolomic biomarkers into current screening protocols necessitates validation through large-cohort studies to confirm their reliability and efficacy. The utilization of automated fecal biomarker profiling systems could facilitate this integration, offering a convenient and efficient approach for screening large populations (Kraemer et al., 2020).

We will explore feasible pathways to develop clinically applicable testing platforms, such as integrated detection systems based on multiplex PCR, NGS panels, mass spectrometry, or microfluidic chips. Critical priorities include streamlining sample processing workflows, enabling automation, controlling costs, and reducing turnaround time (TAT). Concurrently, we will address the need for standardized bioinformatics analysis pipelines and simplified clinical interpretation of results. In future studies, we will isolate and culture key candidate bacterial strains for co-culture validation with normal human colonic epithelial cell lines, adenoma-derived cell lines, or patient-derived colonic organoids. We will specifically assess the direct regulatory effects of these bacteria (or their metabolites/supernatants) on host cell proliferation, apoptosis, migratory capacity, and inflammatory responses. Concurrently, we will monitor dynamic changes in critical metabolites (butyrate, deoxycholic acid) in co-culture systems. Through exogenous supplementation of metabolites (e.g., sodium butyrate) or application of specific inhibitors, we will investigate whether particular metabolites mediate the observed phenotypic effects and delineate their underlying signaling pathways.

## Data Availability

The datasets presented in this study are publicly available. This data can be found here: https://ngdc.cncb.ac.cn/gsa-human, accession number HRA012317.
